# Size Functions for the Morphological Analysis of Melanocytic Lesions

**DOI:** 10.1155/2010/621357

**Published:** 2010-03-14

**Authors:** Massimo Ferri, Ignazio Stanganelli

**Affiliations:** ^1^Department of Mathematics, The Advanced Research Center on Electronic Systems for Information and Communication Technologies E. De Castro (ARCES), University of Bologna, 40126 Bologna, Italy; ^2^Dermatologia Oncologica, Istituto Tumori Romagna (IRST), 47014 Meldola, FC, Italy; ^3^Ospedale Niguarda, 20162 Milano, Italy

## Abstract

Size Functions and Support Vector Machines are used to implement a new automatic classifier of melanocytic lesions. This is mainly based on a qualitative assessment of asymmetry, performed by halving images by several lines through the center of mass, and comparing the two halves in terms of color, mass distribution, and boundary. The program is used, at clinical level, with two thresholds, so that comparison of the two outputs produces a report of low-middle-high risk. Experimental results on 977 images, with cross-validation, are reported.

## 1. Introduction

The incidence of malignant melanoma in fair-skinned patients has increased dramatically in most parts of the world over the past few decades. Because the prognosis of melanoma depends almost entirely on tumor thickness, early detection of thin melanoma is important for the survival of patients [1, 2]. The diagnostic accuracy of the clinical examination of pigmented skin lesions, however, is still rather poor. Literature results arise the evidence that

- the ability of general practitioners to early diagnose CMM with the naked eye is very low;
- the ability of dermatologists to early diagnose CMM with the naked eye ranges from 50% to 75%;
- there is a high rate of false positive (causing unneeded surgical excision).

In the last decade dermoscopy has changed the evaluation of the diagnosis of pigmented skin lesions. Dermoscopy is a noninvasive technique that enables the clinician to perform direct microscopic examination of diagnostic features, not seen by the naked eye, in pigmented skin lesions. This technique is more accurate than naked eye examination for the diagnosis of cutaneous melanoma, in suspicious skin lesions when performed in the clinical setting [3].

A complementary effort is in the automatization of the diagnostic process. Several rather successful computer programs have been implemented to the aim of an automatic analysis of melanocytic lesions and their discrimination between naevi and melanomas (see, e.g., [4–8]; see also [9, 10] for a comparison between automatic and human performance). Most of them keep into account the traditional ABCDE parameters used by dermatologists: Asymmetry (of boundary, texture, and color), Boundary (irregularity and dishomogeneity), Color (presence of several colors), Dimension, and Evolution. In particular, asymmetry is generally based on quantitative comparison of the two parts into which a lesion image is split by its principal axes. Here we focus on asymmetry, perhaps the most important cue. We have developed a new method for comparing in a qualitative, yet precise way the two parts of a lesion at the sides of a splitting line. The mathematical tool for comparison is the theory of Size Functions, applied to three features: boundary shape, mass, and color distribution.

For each splitting line of a pencil we get an asymmetry measure, so forming a map (two for each of the three features). Some characteristic numbers of the six maps are finally fed to a Support Vector Machine. A classification experiment has been led on data set of 977 lesions with very good results. The whole research is a follow-up of the ADAM project of the European Union.

We are well aware that “qualitative measure” reads like an oxymoron; of course, we mean that we compute a precise, objective, repeatable measure of the difference between the two half images; yet, this difference is of a qualitative kind, in that it is not bound to geometric deformations, superimpositions or the like. This is actually the great advantage of using topological and not just geometrical tools.

## 2. Size Functions

Size Functions (SFs) are modular invariants of whatever signal the user is interested in [10]; in the present case, the concerned features are boundary shape, mass, and color distribution. Size functions are maps from the plane to the (extended) natural numbers. They depend on two inputs: an object (e.g., a lesion boundary) and a real map, called measuring function defined on it (e.g., distance from the center of mass). Essentially, the SF registers the behavior of the measuring function by using Morse theory (see [11]). SFs are “qualitative” not only in that they are topological in nature, but also in that a “similarity” based on them depends on the user's choice of a measuring function and of a distance between SFs adapted to the context.

**Figure 1:**
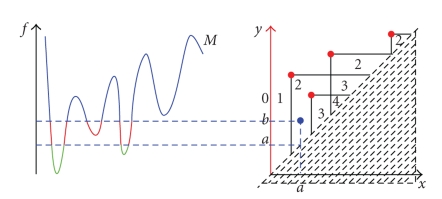
A curve and its Size Function.

Let us recall the definition of an SF, adapted from the more general setting of [12], where measuring functions are allowed a multidimensional range. Consider a continuous real-valued function *φ* : *M* → **R**, defined on a subset *M* of a Euclidean space. The * Size Function* of the pair (*M*, *φ*) is a function *ℓ*_(*M*,*φ*)_ : **R** ^2^ → **N** ∪ {*∞*}. For each pair (*x*, *y*) ∈ **R** ^2^, consider the set *M*_*x*_ = {*P* ∈ *M* : *φ*(*P*) ≤ *x*}. The value *ℓ*_(*M*,*φ*)_(*x*, *y*) is defined to be the number of the connected components of *M*_*y*_ which contain at least one point in *M*_*x*_. The discrete version of the theory substitutes the subsets of the plane with a graph *G* = (*V*, *E*), the function *φ* : *M* → **R** with a function *φ*′ : *V* → **R** and the concept of topological connectedness with the usual connectedness notion for graphs. Figure 1 shows the size function obtained from a curve with the ordinate as measuring function.

## 3. Classification

**Figure 2:**
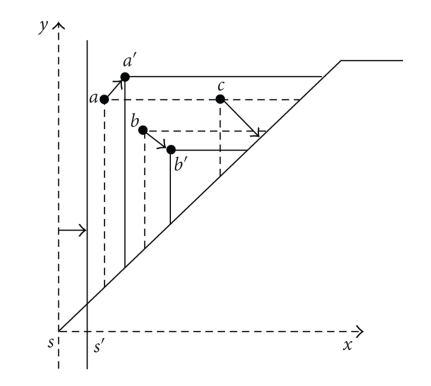
The matching distance.

SFs have a standard structure, the one of superimposed triangles already apparent in Figure 1. This has an important outcome, in that the relevant information can be condensed in the vertices of those triangles [13]. Comparison of two images (as far as the criterion intrinsic to the measuring function is concerned) can then be carried out by comparing the sets of these points. Several distances can be defined on the set of SFs; one which is very successful is the matching distance (see Figure 2).

Distance from templates generally produces numbers of some significance with respect to a classification. Unfortunately, there do not exist archetypal naevi or melanomas, so the task is harder than for classical classification problems. We use distances for measuring asymmetries, as we shall see further on. These distances produce other characteristic numbers. At this point, Statistical Learning comes into play; Vectors of characteristic numbers are the input of a Support Vector Machine.

## 4. Segmentation

**Figure 3:**
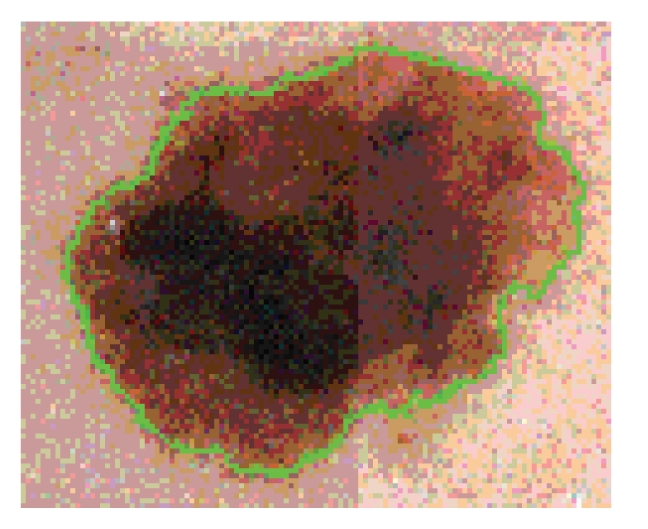
A segmentation example.

The first processing step is segmentation, that is, the isolation of the skin lesion from its background. (See Figure 3; the separating curve is drawn green). This is carried out with well-tested methods depending on several parameters, most of which have been fixed by experiment. Tuning of one of the remaining, permits the removal of most hairs. This is notoriously a serious problem in the processing of dermatological images, and has been solved by the operations of erosion and dilation coming from mathematical morphology.

## 5. Asymmetries

**Figure 4:**
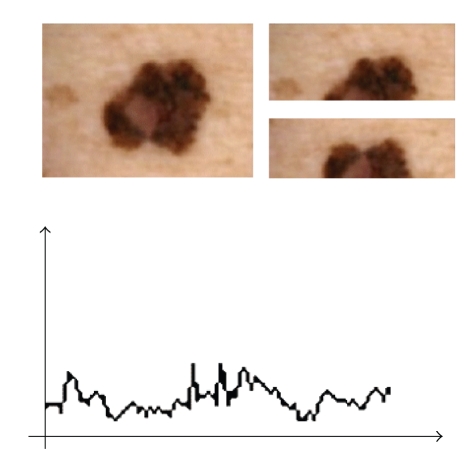
One of the splittings of a lesion and the whole curve of distances.

The experience of dermatologists suggests that a major criterion for suspecting malignancy is the asymmetry of various aspects of the lesion. We have followed this suggestion by splitting each lesion in two halves by a straight line passing through the center of mass. Comparison of the two halves is then performed by computing the distance between their Size Functions. This represents a definite progress with respect to classical methods for detecting asymmetry; these detected only geometrical asymmetry, while distances of Size Functions determine also qualitative asymmetry. We repeat the splitting for 45 equally spaced radial lines, so getting distance as a function of angle (see Figure 4). From this curve the software extracts a set of characteristic numbers: min, max, average, min plus the value at 90° from min, integral, first moment, variation, min derivative, max derivative, integral of absolute value of derivative, and variation of absolute value of derivative. A Support Vector Machine with a third-order kernel is fed with these numbers, computed for each measuring function. Actually, the vectors also contain three more parameters: area, perimeter, and a bumpiness measure coming from the SF of the whole lesion, with distance from center of mass as the measuring function. An initial set of experiments had been carried out with 90 lines instead of 45, but the hit ratio was just slightly higher, while almost doubling computing time.

We have used six measuring functions to distil the structure of boundary, mass distribution, and color distribution, respectively. The first is the distance (of boundary points) from the splitting line. The second sums grey levels along segments orthogonal to the splitting line. The third sums distances of colors (in RGB space) of consecutive pixels along segments orthogonal to the splitting line. Our initial experiments used just these three measuring functions. Adding their three opposite functions improved the hit ratios of 2 to 5 percentage points.

## 6. Experimental Results

The present method has been tested on well-controlled lesion images. The acquisition setup consists of an LEICA 650 M stereomicroscope and a Sony 3CCD-930 color video camera. The illumination of the stereomicroscope consists of a 12 V/50 W halogen lamp that creates a bundle of light perpendicular to the area of interest. The digital images have been archived by means of the DBDERMO Mips software package (Dell'Eva-Burroni, Siena).

Over half of the data set used in the present research, had already been the subject of a formal study of clinical diagnostic validation using also the local population-based cancer registry (i.e., Registro Tumori Romagna) to cross-check for possible false negatives, published on [14]. The data set comes from the daily practice of one of us (Stanganelli); of course, only “interesting” naevi had been acquired. All melanomas and several naevi have been subjected to histological test; all remaining naevi have been subjected to follow-up. We have selected 977 images of melanocytic lesions (melanomas and naevi) acquired in epiluminescence microscopy with a fixed 16-fold magnification. The only selection criterion was that the lesion be entirely visible.

The data set contains 50 melanomas (28 of them with thickness less than 0.75 mm) and 927 naevi. Cross-validation has been performed in three ways. In test H, every second image was assigned to the training set (melanomas were listed consecutively). In tests R1 and R2, a training set of 25 melanomas and 500 naevi was randomized from the data set. The test set was formed by the complement (the remaining 25 melanomas and 427 naevi). A fourth test (S) was performed without cross-validation, with the whole data set both as training and test set; we interpret the not much higher scores of test S as a proof of stability. In Table 1 we report, for each of tests H, R1, R2, and S, the specificity and sensitivity of what we judge to be the best performances.

**Figure 5:**
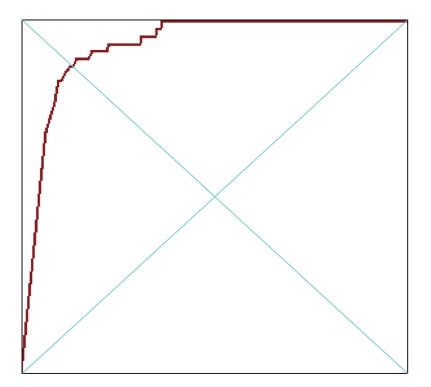
The ROC curve of the single-set S test.

As a further information, in test S a 100% specificity was attained only at cost of 4% sensitivity, but the decrease of specificity to 93.64% yielt a jump to 70% sensitivity. 100% sensitivity was reached at 63.65% specificity. We also report the ROC curve of test S in Figure 5.

Our system is not intended to be provided to the public as a yes/no diagnostic tool; it yields a risk index in the following way. Two classifiers, one tuned at high sensitivity, the other at fairly good specificity, give their response; if they agree to classify the lesion as a naevus (resp., a melanoma) then a low (resp., high) risk is stated; if they disagree, the output is of middle risk. A comparison has been done between the output of this compound classifier and the judgement of an expert dermatologist, who had classified the lesions as sure melanomas, sure naevi and uncertain. The percentages reported in Table 2 refer to the fractions of the three classes (as classified by the human expert) labeled by the machine with the three risk levels.

## 7. Comparison

A true comparison with other research group is problematic. As stressed in [5], there are quite different selection criteria, melanomas/naevi ratios, data set sizes, analysis methods. Instead of reporting selected results of competitors, we refer to Table 1 of that thorough paper. We just would like to comment on very high sensitivity scores (over 95%). With the noticeable exception of Seidenari et al. [4], such scores seem to have been attained either with very small data sets, or with high melanoma percentages, so in situations which appear to be rather far from real-world ones.

Even counting them, the result of our cross-validated test R1 is placed in the top third of the reported scores. Of course, the single-set test S places us at an even higher rank.

It would be interesting to compare—as suggested by a referee—the asymmetry assessment given by our method with the one given by an expert dermatologist. This is unfortunately not possible, since our evaluation does not consist of a single measure, but of 66 (see Section 5), what compelled us to use Support Vector Machines for classification.

In [15] a comparison of the performance of our system and of human operators (three Dermatologists and three General Practictioners) was carried out on a smaller data set of 31 melanomas and 103 naevi. We report the results in Table 3.

## 8. Conclusions

The true novelty of the presented method consists in the use of a qualitative but objective mathematical tool, the Size Functions, to evaluate asymmetry (of boundary, color, and mass distribution). Three experiments with 977 lesions, carried out under cross-validation, show very good performances. Are the results sufficient to make our method definitely preferable to others? No! But its good hit ratio, together with the complete independence from the competitors' tools, make our method a tempting candidate for integration. In this line of thought, comparison aimed to integration should maybe prevail over competition.

## Figures and Tables

**Table 1:** Evaluation of classification results.

	H	R1	R2	S
Specificity	83.84	87.1	86.24	87.16
Sensitivity	84	90	86.67	96.41

**Table 2:** Hit ratio of risk index computation.

	Naevus	Uncertain	Melanoma
Low risk	87.11	51.76	0
Middle risk	10.82	38.82	4.76
High risk	2.06	9.41	95.24

**Table 3:** ELM: Epiluminescence diagnosis (Dermatologists); Clin: Clinical diagnosis (Dermatologists); GP: Clinical diagnosis by General Practitioners; ADAM: our system.

	ELM	Clin	GP	ADAM
Sensitivity	75	74	81	84
Specificity	80	83	73	72
